# Sex-dependent influence of LMAN1 on allergen-induced airway hyperresponsiveness

**DOI:** 10.1093/jimmun/vkaf126

**Published:** 2025-06-15

**Authors:** Lindsay G Swaby, Faith L Sauber, Madelyn H Miller, Mylena C Xavier, Wesley Lim, Peter Daniel Poulson, Xiang Zhu, Bin Zhang, Justine T Tigno-Aranjuez

**Affiliations:** Department of Molecular Pharmacology and Physiology, Morsani College of Medicine, University of South Florida, Tampa, FL, United States; Immunity and Pathogenesis Division, Burnett School of Biomedical Sciences, College of Medicine, University of Central Florida, Orlando, FL, United States; Biotechnology and Immunology Research, Eli Lilly and Company, Indianapolis, IN, United States; Immunity and Pathogenesis Division, Burnett School of Biomedical Sciences, College of Medicine, University of Central Florida, Orlando, FL, United States; Immunity and Pathogenesis Division, Burnett School of Biomedical Sciences, College of Medicine, University of Central Florida, Orlando, FL, United States; Immunity and Pathogenesis Division, Burnett School of Biomedical Sciences, College of Medicine, University of Central Florida, Orlando, FL, United States; Immunity and Pathogenesis Division, Burnett School of Biomedical Sciences, College of Medicine, University of Central Florida, Orlando, FL, United States; Department of Genomic Medicine, Lerner Research Institute, Cleveland Clinic, Cleveland, OH, United States; Immunity and Pathogenesis Division, Burnett School of Biomedical Sciences, College of Medicine, University of Central Florida, Orlando, FL, United States

**Keywords:** LMAN1, ERGIC-53, airway hyperresponsiveness, HDM, asthma

## Abstract

Allergic asthma is a chronic inflammatory disease of the airways characterized by a type 2-high adaptive immune response towards common aeroantigens such as dust mite, pollen, and animal dander. Despite the advances made toward translation of various biologics into the clinic, the limited efficacy of these therapies in certain populations, combined with the ineligibility of some patients for treatment (clinically or economically), have led to the continued need for the development of more widely effective allergic asthma therapies. Our lab previously identified lectin mannose-binding 1 (LMAN1) as a novel receptor for house dust mite (HDM) and showed that *in vitro,* LMAN1 downregulated inflammatory NF-κB signaling in DCs in response to HDM. In this follow-up work, we investigated the *in vivo* relevance of LMAN1 by subjecting LMAN1 knockout (KO) mice and wild type (WT) littermate controls to a model of HDM-induced allergic asthma. Surprisingly, we discovered that loss of LMAN1 led to opposing effects on airway hyperresponsiveness (AHR), which were dependent on the sex of the mice. HDM-treated female LMAN1 KO mice showed increased AHR, while HDM-treated male KO mice showed decreased AHR, compared with their WT counterparts. We further identified the features of HDM-induced asthma which may account for the gender-biased effects of LMAN1 on lung function. This work not only highlights the complexity of the loss of LMAN1 in vivo but also suggests that such sex-dependent responses should be taken into consideration when pursuing LMAN1 as a therapeutic target for treatment of allergic asthma.

## Introduction

Asthma is a chronic inflammatory disease currently affecting at least 350 million people worldwide, including an estimated 13.4% of adults and 11.6% of children in the United States.^1^ Allergic asthma, the most common asthma subset, is characterized by a type 2 high adaptive immune response toward aeroallergens such as dust mite, molds, pollen, and animal dander. While corticosteroids and β2-adrenoreceptor agonists comprise the mainstay asthma therapies, these medications cannot sufficiently manage symptoms in patients with severe refractory asthma, and 5-10% of asthma patients suffer from uncontrolled asthma that is associated with reduced quality of life, significant financial burden, and mortality and morbidity.^2^^,^^3^ Recently, various biologics have been developed to target specific components of the Th2 response in severe asthma, with well-known examples including Omalizumab, which targets IgE; Mepolizumab and Reslizumab, which target IL-5; Benralizumab, which targets the IL-5 receptor; and Dupilumab, which inhibits IL-4 and IL-13.^4^ However, these biologics are costly, effective only in a subset of patients, and restricted based on clinical criteria, highlighting the need for more widely available allergic asthma therapies.^5^

The dendritic cell (DC) is a promising target for the treatment of allergic asthma due to its ability to modulate the downstream Th2 response. During initiation of the type 2 response, airway epithelial cells (AECs) release cytokines known as “alarmins,” including TSLP, IL-25, and IL-33, which initiate priming or conditioning of DCs.^6^^,^^7^ These primed DCs induce polarization of type-2 T helper cells (Th2) at regional lymph nodes, and the Th2 cytokines IL-4, IL-5, and IL-13 subsequently promote asthma symptoms by inducing inflammatory cell recruitment, IgE production, excess mucus secretion, and airway smooth muscle cell contraction. Over time, the persistent Th2 response leads to airway remodeling characterized by collagen deposition and fibrosis, thickening of the smooth muscle layer, and dysregulation of the epithelial barrier.^8^^,^^9^ As these multiple events occur downstream of DCs, targeting the DCs can potentially prevent the Th2 response and airway remodeling. The efficacy of DCs as a therapeutic target is evidenced by the success of the recently-developed biologic Tezepelumab, which inhibits TSLP and therefore prevents the priming of DCs towards a type 2 phenotype.^10^^,^^11^ With these in mind, our lab recently reported Lectin mannose-binding 1 (LMAN1) as a novel receptor for house dust mite (HDM) allergen on DCs.^12^ We showed in vitro that LMAN1 downregulates inflammatory signaling in response to HDM and that LMAN1 cell surface expression is reduced on the peripheral DCs of asthmatics. While these findings suggested that LMAN1 could potentially be an effective therapeutic target, a better understanding of its role in allergic asthma was needed.

In the current work, we investigate the *in vivo* relevance of LMAN1 in a murine model of HDM-induced allergic asthma. We identify a role for LMAN1 in influencing AHR in a sexually dimorphic manner.

## Materials and methods

### Mouse strains

LMAN1 knockout (KO) mice on a C57BL/6J background were kindly provided by Dr Bin Zhang.^13^^,^^14^ Mice were shipped to Texas A&M Institute for Genomic Medicine (TIGM) for rederivation before establishment of a local LMAN1 KO mouse line at the University of Central Florida. LMAN1 mice were maintained by heterozygous breeding, and WT and LMAN1 KO littermates were used for experiments. The mice were housed in the Burnett School of Biomedical Sciences Lake Nona Animal Facility in standard microisolator caging with *ad libitum* access to food and water. All procedures were conducted with the approval of the Institutional Animal Care and Use Committee at the University of Central Florida.

### Intratracheal HDM asthma model

Intratracheal instillations were performed in eight to eleven-week-old mice. Mice were anesthetized with isoflurane, and 3% lidocaine was administered orally as a topical anesthetic. An otoscope and guide needle were used to insert an intratracheal cannula, and *Dermatophagoides farinae* extract (Greer Laboratories; Lenoir, North Carolina, USA) was delivered with a gas-tight syringe. On day 0, each mouse received 60 µg extract. On days 7, 8, 9, 10, and 11, each mouse received 12.5 µg extract. Mice were sacrificed on day 14.

### FlexiVent measurements

On day 14, mice were anesthetized by intraperitoneal injection of a cocktail containing Ketamine, Xylazine, and Acepromazine. Mice were then confirmed to be unresponsive and were cannulated through the trachea and connected to the FlexiVent ventilator system (Scireq; Montreal, Canada). Baseline readings of respiratory mechanics were obtained using the Deep Inflation, SnapShot-150, Quick Prime-3, and PVs-P functions. Increasing doses of inhaled methacholine (3.125, 6.25, 12.5, 25, 50, and 100 mg/ml) were administered to the mouse using the Aeroneb, and measurements at each dose were assessed using 12 iterations of SnapShot-150 and 12 iterations of QuickPrime-3.

### Lung histological scoring

After FlexiVent measurements, mice were euthanized, and serum and bronchoalveolar lavage were collected. After the collection of all other needed tissues, the lungs were inflated and fixed with 10% buffered formalin. Fixed samples were embedded in paraffin and stained with hematoxylin and eosin (H&E), periodic acid Schiff (PAS) and trichrome blue. H&E tissue sections were scored blindly as previously described.^15^ Scores for bronchoarterial inflammation, amuscular blood vessel inflammation, intralveolar space inflammation, pulmonary vein inflammation, and pleural inflammation were combined into an Inflammatory Index score with a maximum of 16. For PAS sections, both the incidence and severity of mucus production were scored blindly for a maximum score of 4. Trichrome sections were scored blindly for collagen deposition as previously described.^15^^,^^16^ Peribronchial and perivascular trichrome staining was scored for intensity on a scale of 0–3, while parenchymal trichrome staining was scored for intensity from 0 to 3. These features were combined for a maximum score of 6. To determine epithelial thickness and lumen area, 10 bronchioles with a diameter <400 µm were measured.

### Flow cytometry

After sacrifice on day 14, mice were cannulated through the trachea, and bronchoalveolar lavage (BAL) was performed by flushing the lungs three times with 0.5 ml of 0.6 mM EDTA in PBS. The recovered solution was spun down, and cells were resuspended in FACS buffer (PBS with 1% FBS) for counting. One million cells were stained with the following antibodies: FITC anti-mouse CD3 (145-2C11, BioLegend; San Diego, California, USA), FITC anti-mouse B220 (RA3-6B2, BioLegend; San Diego, California, USA), PE anti-mouse Siglec F (E50-2440, BD Biosciences), PerCP anti-mouse CD11b (M1/70, BioLegend; San Diego, CA), PE-Cy7 anti-mouse Ly-6G (1A8, BioLegend: San Diego, California, USA), APC anti-mouse CD11c (N418, Thermo Fisher; Waltham, MA), and APC-Cy7 anti-mouse CD45 (30-F11, BioLegend; San Diego, California, USA). Gating was performed as previously described.^15^

For ICS staining, one lung lobe was collected and homogenized using a gentleMACS Lung Dissociation Kit and a gentleMACS Dissociator (Miltenyi Biotec; Bergisch Gladbach, Germany) according to the manufacturer’s instructions. Two million lung cells were incubated at 37 °C for 4 h with 5 ng/ml PMA, 0.5 μg/ml ionomycin, and protein transport inhibitors (GolgiPlug and GolgiStop, BD Biosciences; San Jose, California, USA). After stimulation, cell surface staining was performed using APC-Cy7 anti-mouse CD45 (30-F11, BioLegend; San Diego, CA) and FITC anti-mouse CD4 (GK1.5, BioLegend; San Diego, California, USA). Cells were then fixed and permeabilized using a Fixation/Permeabilization kit (BD Biosciences) and were stained intracellularly using PerCP/Cy5.5 anti-mouse IL-4 (11B11, BioLegend; San Diego, California, USA), PE anti-mouse IL-5 (TRFK5, BioLegend: San Diego, California, USA), and APC anti-mouse IL-17a (TC11–18H10.1, BioLegend; San Diego, California, USA). Gating was performed as previously described.^15^

ILC2 staining was performed on two million lung cells obtained by gentleMACS homogenization as described above. The following antibodies were used: FITC anti-mouse lineage (CD3, Gr-1, CD11b, B220, Ter-119) (BioLegend; San Diego, California, USA), FITC anti-mouse TCRβ (H57– 597, BioLegend: San Diego, CA), FITC anti-mouse TCRδγ (eBioGL3, Thermo Fisher; Waltham, MA), FITC anti-mouse CD11c (N418, BioLegend; San Diego, California, USA), FITC anti-mouse NK1.1 (PK136, BioLegend; San Diego, California, USA), APC anti-mouse CD45 (30- F11, BioLegend; San Diego, CA), APC/Cy7 anti-mouse KLRG1 (2F1, BioLegend; San Diego, California, USA), PerCP anti-mouse Thy1.2 (53–2.1, BioLegend; San Diego, California, USA), PE anti-mouse ST2 (RMST2–2, Thermo Fisher; Waltham, MA), and PE-Cy7 Sca-1 (E13–161.7, BioLegend; San Diego, California, USA). The gating strategy was performed as previously described.^15^ A lineage^-^CD45^+^ gate was identified using a sample stained with CD45 and lineage isotype control. Lineage^-^CD45^+^ cells were then gated to identify ILC2 populations.

Samples were acquired using the NovoCyte cytometer (Agilent Technologies; Santa Clara, California, USA), and analysis was performed in NovoExpress software.

### Serum IgG_1_ measurement

HDM-specific IgG_1_ was measured by direct ELISA using Nunc Maxisorp plates (Thermo Fisher; Waltham, Massachusetts, USA). Plates were coated overnight at 4 °C with *D. farinae* extract (Greer Laboratories; Lenoir, North Carolina, USA), followed by blocking for 1 h with 5% BSA in PBS. Serum samples were diluted 1:50 in PBS-5% BSA-0.025% Tween-20, and 100 µl of each diluted sample was added to each well and incubated overnight at 4 °C. The next day, plates were incubated with biotin anti-IgG_1_ (BioLegend; San Diego, California, USA) for 4 h at room temperature, followed by incubation with streptavidin-HRP (BioLegend; San Diego, California, USA) for 30 min. Plates were developed with TMB substrate. After addition of stop solution, absorbance was read at 450 nm using a Biotek plate reader (Agilent Technologies; Santa Clara, California, USA).

### Cytokine and chemokine analysis

For cytokine and chemokine analysis, one lobe of the right lung was collected and homogenized in T-PER buffer (Thermo Fisher; Waltham, Massachusetts, USA). Lysates were standardized to 0.75 mg/ml prior to using in a Legendplex Mouse Inflammation or Mouse Th bead-based multiplex assay (BioLegend; San Diego, California, USA). Samples were acquired using the NovoCyte flow cytometer (Agilent Technologies; Santa Clara, California, USA), and analysis was performed using the Legendplex analysis software (BioLegend; San Diego, California, USA). Additional ELISAs for mouse IL-33, IL-25 and TSLP were performed as per manufacturer’s directions (BioLegend; San Diego, California, USA).

### Statistical analysis

Statistical analysis was performed using Prism 10 (Graphpad, San Diego, California, USA). An unpaired Student’s t-test was used for comparison of two groups and a two-way ANOVA followed by post hoc tests was used for all other comparisons. Data are expressed as means ± SEM. Numbers of subjects per experimental group and additional details are indicated in the legend for each figure.

## Results

### LMAN1 influences HDM-induced AHR in a sex-dependent manner

**Figure 1.**
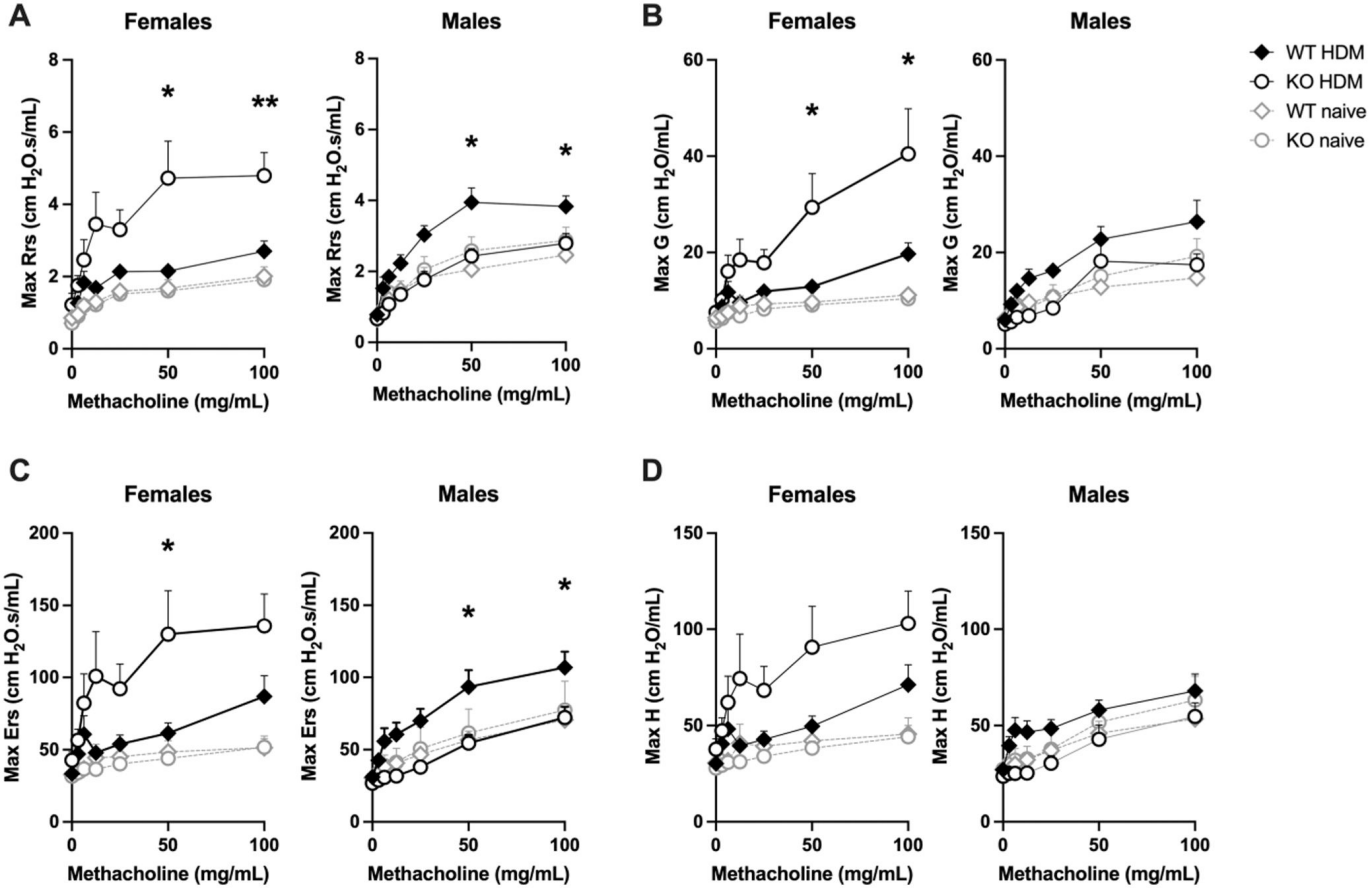
LMAN1 influences HDM-induced AHR in a sex-dependent manner. WT and LMAN1 KO mice were left untreated or were subjected to an HDM-induced asthma model. On day 14, mice were anesthetized, intratracheally cannulated, and connected to the FlexiVent ventilator. Increasing doses of methacholine were administered to the nebulizer, and measurements were obtained of (A) maximum respiratory system resistance (Rrs), (B) tissue damping (G), (C) respiratory system elastance (Ers), and (D) tissue elastance (H). Data represent mean ± SEM. A Student *T*-test was used to assess significance at each dose of methacholine. Aggregated data from multiple experiments is shown with n = 7-13 per group. *P < 0.05, **P < 0.01.

Airway hyperresponsiveness (AHR), or excessive narrowing of the airways in response to stimuli, is a cardinal feature of asthma. To investigate the role of LMAN1 in allergen-induced AHR, we subjected wild-type (WT) and LMAN1 knockout (KO) mice to a HDM asthma model. On day 14, we evaluated lung mechanics and AHR using the FlexiVent system. Mice were exposed to increasing doses of methacholine and maximum airway resistance (Rrs), tissue damping (G), respiratory system elastance (Ers), and tissue elastance (H) were measured as done previously.^15^^,^^17^ Having shown that LMAN1 exerted an inhibitory effect on the response to HDM in vitro*,^12^* we hypothesized that the absence of LMAN1 *in vivo* would lead to an enhanced response to HDM and worsened AHR. Surprisingly, when the data were stratified, we observed opposing effects of LMAN1 on AHR which depended on the sex of the mice. In HDM-treated females, loss of LMAN1 led to a significant increase in maximal Rrs, Ers and G for the highest methacholine doses when compared with WT mice (Fig. 1A–D). Conversely, HDM-treated male LMAN1 KO mice showed a significant reduction in maximal Rrs and Ers compared with their WT counterparts (Fig. 1A–D). No significant baseline differences were observed between WT and LMAN1 KO naïve mice for multiple parameters of lung function measured, indicating no anatomical or structural defects which might result in the observed differences (Fig. S1A). However, HDM treatment did cause a significant sex-dependent alteration of baseline lung function, promoting a significant *increase* in Rrs in KO vs WT females and a significant *decrease* in baseline G and Ers in KO vs WT males (Fig. S1B). Taken together, our data suggest that LMAN1 influences HDM-induced AHR in a sex-dependent manner, with loss of LMAN1 worsening AHR in females and improving AHR in males.

### Loss of LMAN1 in males reduces airway inflammation in an HDM asthma model

**Figure 2.**
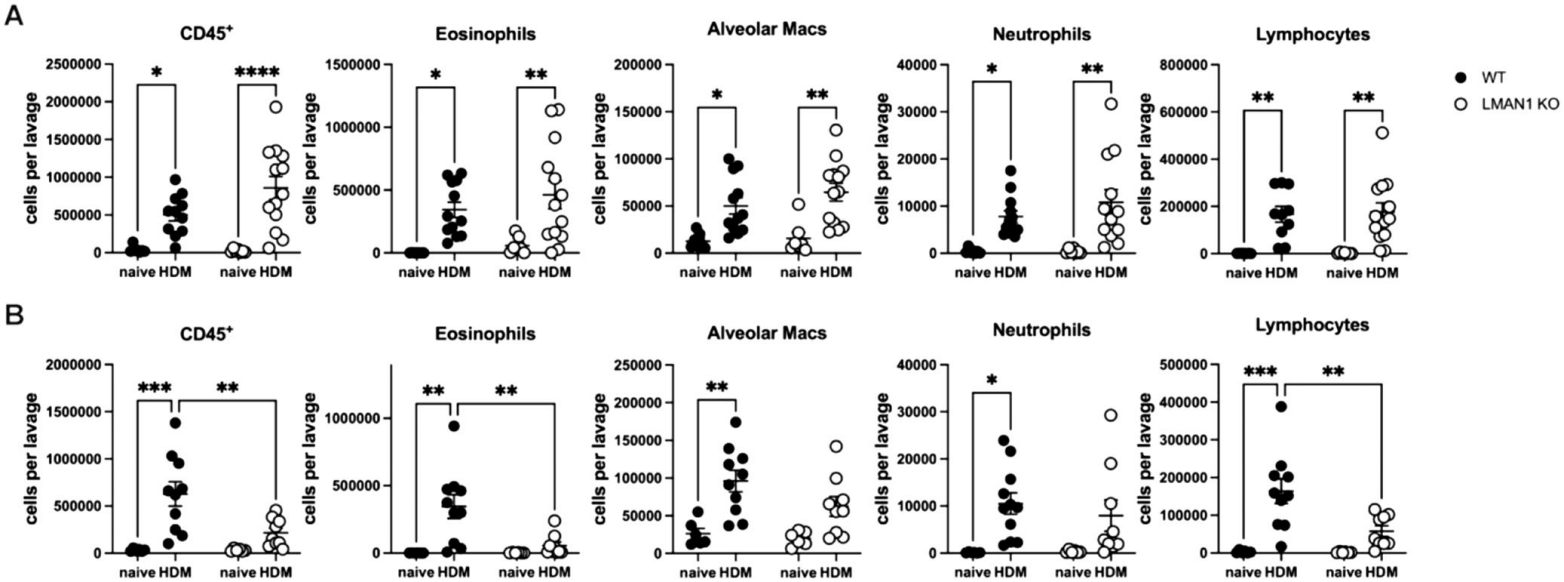
Loss of LMAN1 in males reduces airway inflammation in an HDM asthma model. WT and LMAN1 KO mice were left untreated or were subjected to an HDM-induced asthma model. Mice were euthanized on day 14 and bronchoalveolar lavage (BAL) samples from (A) female mice and (B) male mice were analyzed via flow cytometry. Lines behind scatterplots represent means ± SEM. Results were analyzed using two-way ANOVA with multiple comparisons. Aggregated data from multiple experiments is shown with n = 6 to 13 mice per group. *P < 0.05, **P < 0.01, ***P < 0.001.

Increased airway inflammation has often been implicated in promoting increased AHR.^18–22^ In order to determine whether the observed sex-dependent effects of LMAN1 on AHR are a result of sex-dependent differences in airway inflammation, we collected bronchoalveolar lavage (BAL) cells from naïve or HDM-treated WT and LMAN1 KO mice and performed flow cytometric analysis of immune cell populations using previously described panels and gating strategies^15^^,^^17^ (Fig. S2). We observed that HDM-treated male mice lacking LMAN1 had a significant reduction in the numbers of CD45^+^ cells, eosinophils and lymphocytes when compared to HDM-treated WT mice (Fig. 2B). Interestingly, no significant associations for inflammatory cell recruitment were observed when comparing HDM-treated female WT and LMAN1 KO mice (Fig. 2A). These data suggest that LMAN1 plays a role in promoting HDM-induced airway inflammation in male but not in female mice.

### Male LMAN1 KO mice exhibit decreased lung pathology and lumen narrowing

**Figure 3.**
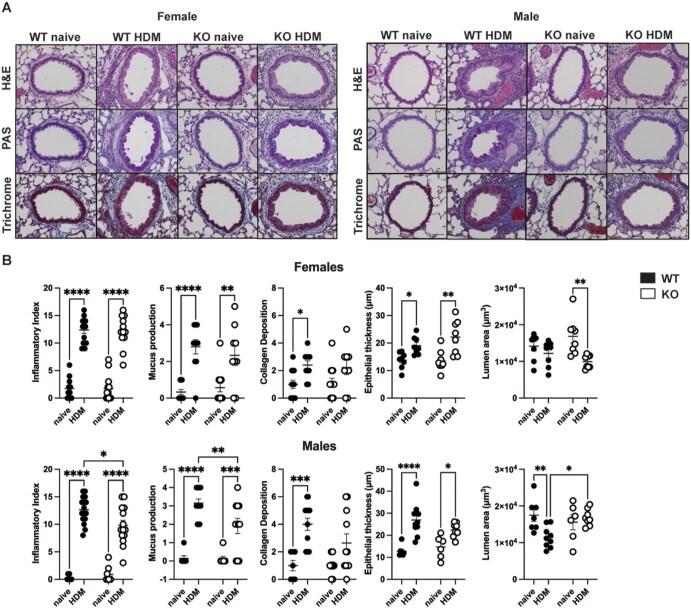
Male LMAN1 KO mice exhibit decreased lung pathology and lumen narrowing. WT and LMAN1 KO mice were left untreated or were subjected to an HDM-induced asthma model. On day 14, mice were euthanized, and lungs were inflated and fixed in formalin. Lung samples were stained with H&E, Periodic Acid Schiff (PAS), or trichrome blue. (A) Representative 40x images of bronchioles in female mice (left) and male mice (right) are shown. (B) Lung inflammatory index was scored for a maximum score of 16, mucus production for a maximum of 4, and collagen deposition for a maximum of 6. Lumen area and epithelial thickness were also measured. Lines behind scatterplots represent means ± SEM. Results were analyzed by two-way ANOVA with multiple comparisons. Aggregated data from multiple experiments are shown with n = 7 to 14. *P < 0.05, **P < 0.01, ***P < 0.001, ****P < 0.0001.

While BAL analysis can give information on inflammatory cell recruitment to the lung, it does not provide information about the specific anatomical location of such inflammation or about HDM-induced structural changes in the airway. To this end, we evaluated HDM-induced lung pathology in naïve or HDM-treated WT and LMAN1 KO mice using previously developed scoring systems^15^^,^^17^ (Fig. S3). Lung tissue was embedded in paraffin and stained with H&E, PAS, and trichrome blue. H&E sections were blindly scored for features of bronchoarterial inflammation, amuscular blood vessel inflammation, intralveolar space inflammation, pleural inflammation, and pulmonary vein inflammation, all of which were combined into one inflammatory index with a maximum score of 16. PAS-stained slides were used for assessment of mucus metaplasia/goblet cell hyperplasia and trichrome blue-stained slides were used for assessment of collagen deposition/fibrosis. Representative lung sections for each group are shown in Fig. 3A and histopathological scoring indices are summarized in Fig. 3B. We observed that HDM-treated male LMAN1 KO mice exhibited significantly reduced inflammatory index and mucus metaplasia scores compared to HDM-treated male WT mice while no difference was observed for the corresponding female groups (Fig. 3A, B). No sex-dependent differences were observed for collagen deposition (Fig. 3A, B). When we performed measurements of lumen area and epithelial thickness, we found that HDM-treated male LMAN1 KO mice had a significantly increased (improved) lumen area compared to HDM-treated male WT mice while no such difference was observed for the corresponding female groups (Fig. 3A, B). No sex-dependent differences were observed for epithelial thickness (Fig. 3A, B). Thus, HDM-treated LMAN1 KO males, but not LMAN1 KO females, exhibited reduced inflammatory pathology and increased (improved) lumen area compared to their WT counterparts.

### Loss of LMAN1 impacts Th2 polarization in male but not female mice

**Figure 4.**
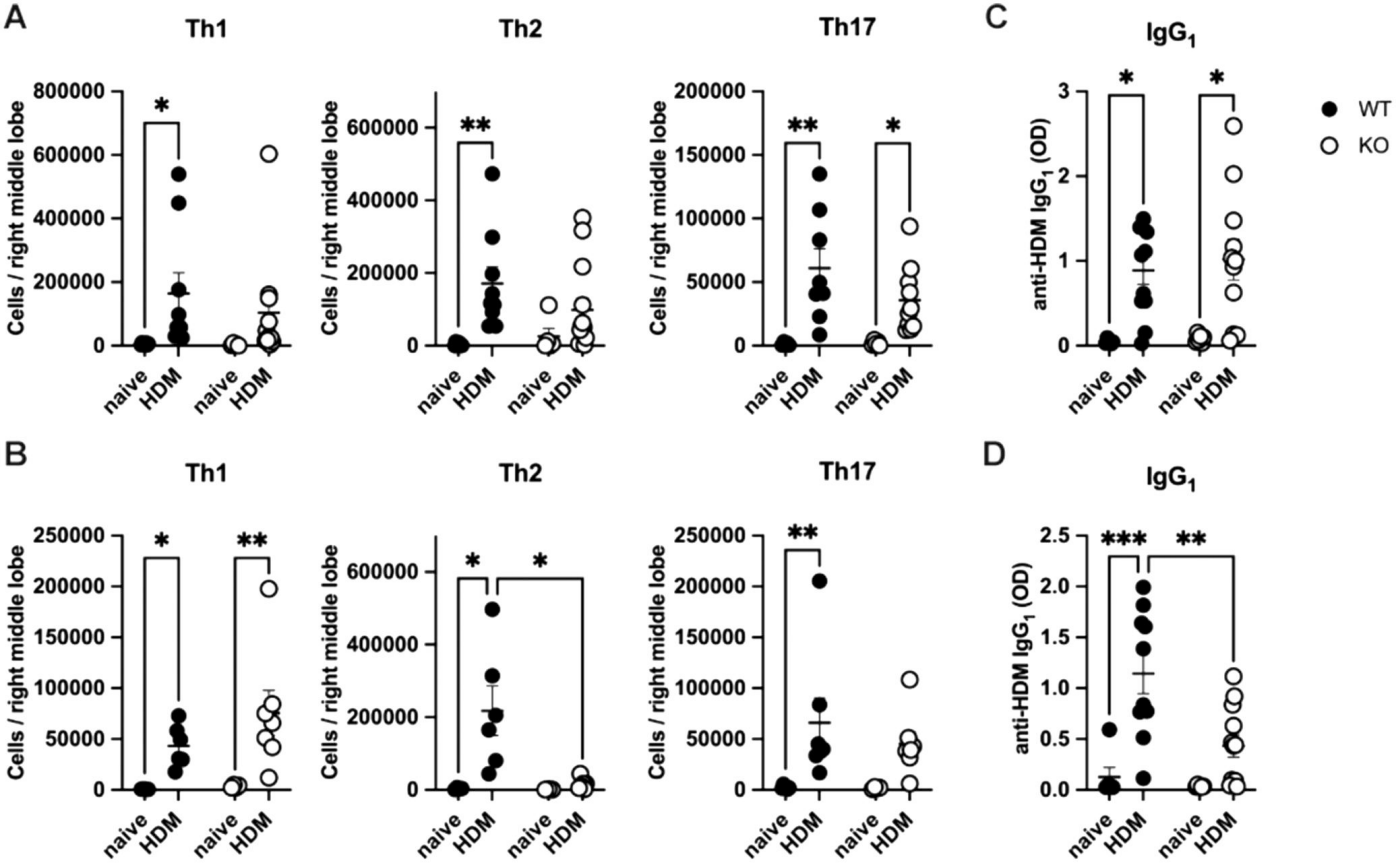
Male LMAN1 KO mice show reduced Th2 polarization. WT and LMAN1 KO mice were left untreated or were subjected to an HDM-induced asthma model. On day 14, mice were euthanized for the collection of lungs and serum. Lungs were enzymatically digested and mechanically homogenized to obtain single-cell suspensions, and intracellular cytokine staining (ICS) was performed for females (A) and males (B). ELISA was used to measure HDM-specific IgG1 in serum obtained from female (C) and male (D) mice. Lines behind scatterplots represent means ± SEM. Results were analyzed by two-way ANOVA with multiple comparisons. Aggregated data from multiple experiments is shown with n = 3 to 15 mice per group for ICS and n = 6 to 12 mice per group for ELISA. *P < 0.05, **P < 0.01, ***P < 0.001.

Other parameters that have been shown to associate with AHR in a sex-specific manner are markers of type 2 inflammation. We therefore questioned whether LMAN1 could modulate divergent responses in males and females by influencing lung CD4^+^ T cell polarization. To investigate this, we performed intracellular cytokine staining (ICS) on naïve and HDM-treated, WT and LMAN1 KO mice of both sexes. Dissociated lung cells were surface-stained with CD45 and CD4 and stimulated for 4 h in the presence of PMA and ionomycin plus transport inhibitors. This was followed by intracellular cytokine staining to identify Th2 cells (CD4^+^IL-5^+^), Th1 cells (CD4^+^IFNγ^+^), and Th17 cells (CD4^+^IL-17^+^) as described previously^15^^,^^17^ (gating strategy shown in Fig. S4). We observed a significant decrease in the numbers of Th2 cells in HDM-treated male LMAN1 KO mice when compared to their HDM-treated WT counterparts (Fig. 4B). This was not observed in female mice (Fig. 4A). Additionally, no differences were observed in Th1 and Th17 responses when comparing HDM-treated LMAN1 KO mice to HDM-treated WT mice of either sex (Fig. 4A, B). We additionally assessed type 2-associated IgG_1_ responses and demonstrated a significant reduction in HDM-specific IgG_1_ responses in HDM-treated male LMAN1 KO mice when compared to their HDM-treated WT counterparts (Fig. 4D). This was not observed in female mice (Fig. 4C). Finally, we also examined Th cytokines present in standardized lung homogenates using a multiplex bead-based assay (Fig. S5). A significant reduction in IL-4 and increase in TNF-α was observed in HDM-treated male LMAN1 KO mice compared to HDM-treated male WT mice (Fig. S5). Other cytokines that were significantly altered included IL-5 and IL-6, which were decreased in HDM-treated male WT mice compared to HDM-treated female WT mice, and IL-9, which was decreased in HDM-treated male KO mice compared to HDM-treated female KO mice (Fig. S5). Overall, these data point to a role for LMAN1 in impacting HDM-induced type 2 immunity in male but not in female mice.

### LMAN1 does not influence lung ILC2 recruitment

**Figure 5.**
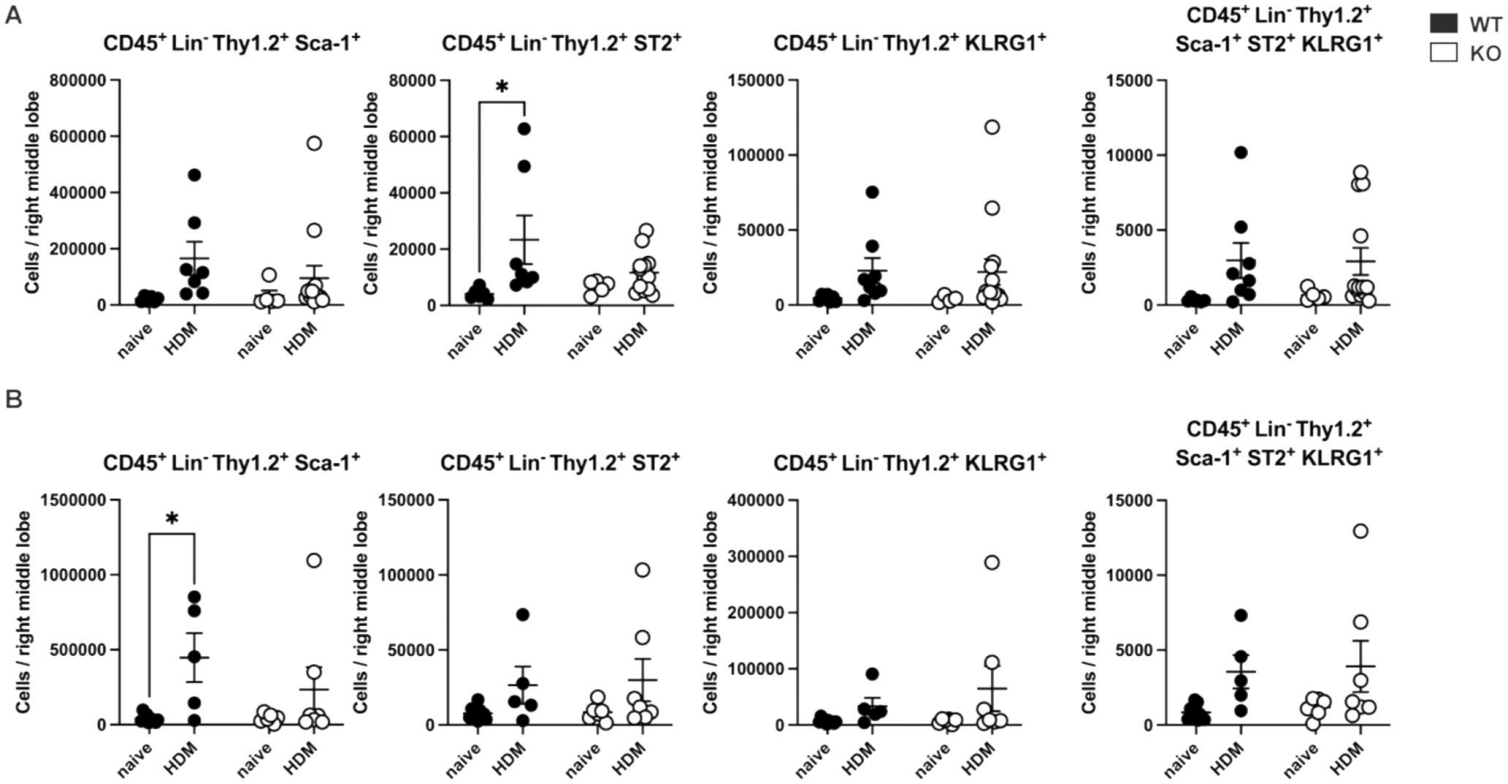
LMAN1 does not influence lung ILC2 recruitment. WT and LMAN1 KO mice were left untreated or were subjected to an HDM-induced asthma model. On day 14, mice were euthanized and lungs were enzymatically digested and mechanically homogenized to obtain single- cell suspensions for flow cytometric identification of ILC2s. Results are shown for CD45^+^ Lin^-^Thy1.2^+^ Sca-1^+^, CD45^+^ Lin^-^ Thy1.2^+^ ST2^+^, CD45^+^ Lin^-^ Thy1.2^+^ KLRG1^+^, and CD45^+^ Lin^-^ Thy1.2^+^ Sca-1^+^ ST2^+^ KLRG1^+^ ILC2 cells in female (A) and male (B) mice. Lines behind scatterplots represent means ± SEM. Results were analyzed by 2–-way ANOVA with multiple comparisons. Aggregated data from multiple experiments are shown with n = 4 to 14 per group *P < 0.05.

As research in both humans and mice has indicated sex-dependent differences in ILC2 responses^23^ and ILC2s have also been implicated in promoting AHR,^24^ we investigated whether LMAN1 might influence lung ILC2 recruitment in a sex-dependent manner. We assessed ILC2 numbers using flow cytometric analysis of enzymatically dissociated lung cells from naïve and HDM-treated WT and LMAN1 KO mice as described previously.^15^ ILC2 cells were defined as Lin^-^ Thy1.2^+^ Sca-1^+^, Lin^-^ Thy1.2^+^ ST2^+^, Lin^-^ Thy1.2^+^ KLRG1^+^, or Lin^-^ Thy1.2^+^ Sca-1^+^ ST2^+^ KLRG1^+^ (refer to gating strategy shown in Fig. S6). In WT females, HDM-treatment increased numbers of Lin^-^ Thy1.2^+^ ST2^+^ ILC2s (Fig. 5A). while, for WT males, HDM-treatment increased numbers of Lin^-^ Thy1.2^+^ Sca-1^+^ ILC2s (Fig. 5B). No other significant differences were observed for the various ILC2 groups for WT and LMAN1 KO mice of either sex (Fig. 5).

### Impact of LMAN1 on the airway epithelium and early inflammatory cytokine production

**Figure 6.**
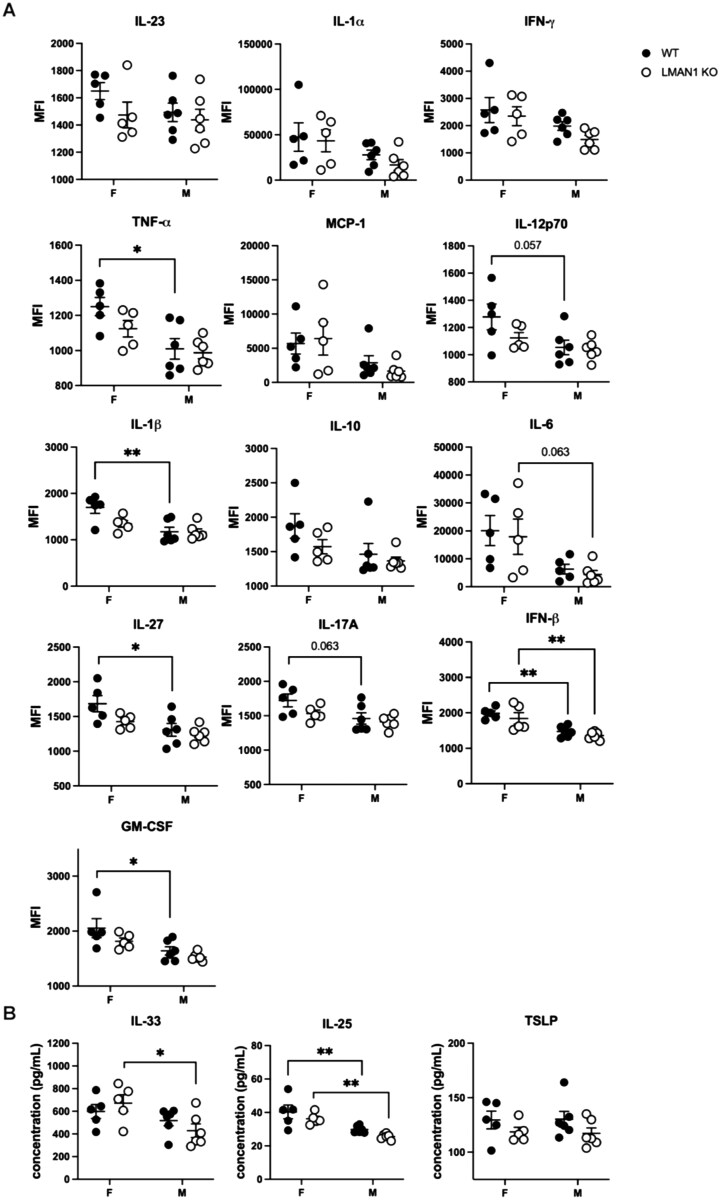
Sex-specific differences in the early production of inflammatory cytokines in WT and LMAN1 KO HDM-treated mice. Male and female WT and LMAN1 KO mice were exposed to HDM intratracheally. On day 1 after exposure, mice were euthanized and lungs were homogenized in cell lysis buffer with protease inhibitors. Cell lysates were standardized and subjected to a multiplex bead-based assay (A) or ELISAs (B) to determine the production of proinflammatory cytokines. Lines behind scatterplots represent means *±* SEM. Results were analyzed by 2-way ANOVA with multiple comparisons. Aggregated data from multiple experiments are shown with n = 5 to 6 per group *P < 0.05, **P < 0.01.

Given the disparity we have been observing in female LMAN1 KO mice regarding worsened AHR without a significant increase in various measures of inflammation, we decided to investigate the contribution of LMAN1 to the early production of inflammatory cytokines. Male and female WT and LMAN1 KO mice were treated with HDM i.t. and euthanized 1 d later to assess inflammatory cytokines produced early in the immune response. Lungs were isolated, homogenized and standardized for assessment of inflammatory cytokine production via bead-based multiplex assay (Fig. 6A) and additional ELISA assays (Fig. 6B). There was an overall reduction in the levels of TNF-α, IL-1β, IL-27, IFN-β, IL-25 and GM-CSF in HDM-treated male WT mice versus HDM-treated female WT mice (Fig. 6). There was a reduction in the levels of IFN-β, IL-33 and IL-25 in HDM-treated male KO mice versus HDM-treated female KO mice (Fig. 6). Given the opposing effects of sex and genotype on AHR, we were particularly interested in mediators that mimicked these directions. While not significant, there was a trend for an increase in the levels of IL-33 in HDM-treated female KO mice versus HDM-treated female WT mice and an opposite trend for a decrease in the levels of IL-33 in HDM-treated male KO mice versus HDM-treated male WT mice (Fig. 6).

Suspecting that the airway epithelium would be a large contributor to the observed effects, we established primary murine tracheal epithelial cell cultures (MTECs) from male and female mice of both genotypes as described previously.^15^ After full differentiation under an air-liquid interface, cells were left untreated or treated with HDM extract overnight prior to isolation of RNA for RNA sequencing. Hierarchical clustering of all groups and volcano plots of pairwise comparisons are shown in Fig. S7. Comparison of top 5 differentially expressed genes (DEGs) from naive M WT and F WT MTECs or from naïve M KO and F KO MTECs, resulted in the identification of Ddx3y, Eif2s3y, Kdm5d, Uty, and Xist, which are known to be expressed in a sex-specific manner (Fig. S7B and D). In order to identify potential pathways that could explain the opposing effects of sex and genotype on AHR, K-means clustering was performed followed by enrichment analysis using KEGG pathways. The top 10 pathways within each cluster are shown in Fig. S8. Some notable KEGG pathways with known impacts on AHR and which are changed in a sex-specific manner in WT mice include relaxin signaling and PI3K-Akt signaling (Fig. S8A), while steroid hormone biosynthesis and adrenergic signaling are altered in a sex-specific manner in KO mice (Fig. S8B).

## Discussion

We showed that loss of LMAN1 influences airway hyperresponsiveness (AHR) in a sex-dependent manner in response to HDM. The occurrence of sex-dependent trends in asthma incidence and severity is well established, with adult females demonstrating a higher incidence of asthma as well as increased asthma-related hospitalizations and death compared with adult males.^25–28^ This trend is often linked with gender differences in hormone levels. Estrogen in females exerts a Th2-enhancing effect, which is thought to be responsible for higher ILC2 levels, enhanced Th2 and Th17 polarization, upregulated histamine receptor expression, and increased cysteinyl leukotriene production seen in females versus males.^29^ Conversely, testosterone provides a protective effect in males by causing a shift towards Th1 immunity.^30–32^ Other gender differences in asthma appear to be genetic, as various single-nucleotide polymorphisms (SNPs) have been linked with sex-specific trends in pulmonary function and asthma risk.^33^

In this work, we investigated the role of LMAN1 in a physiologically relevant mouse model of allergic asthma and showed that LMAN1 regulates both AHR and the inflammatory response to HDM in a sex-dependent manner. Male LMAN1 KO mice showed reduced AHR and an attenuated inflammatory response, as evidenced by trends in lung immune cell infiltration, lung histological scores, serum IgG1 levels, lung CD4^+^ T cell polarization, and lung cytokine levels. These findings suggest that LMAN1 promotes the response to HDM in male mice, a surprising result given our previous finding that LMAN1 acts as a negative regulator *in vitro*. However, it should be noted that our *in vitro* experiments were primarily focused on bone marrow-derived dendritic cells and dendritic cell lines and did not control for the sex of the mice. Our findings furthermore raised the question of whether LMAN1 may act as a positive regulator of the response to HDM in certain cell types. Although our previous *in vitro* experiments focused on the role of LMAN1 in DCs, we have also reported LMAN1 to be expressed on the surface of other cells, including B cells, T cells, neutrophils, and airway epithelial cells, in which it could potentially play a different role, leading to a complex interplay of factors in the in vivo environment.

While LMAN1 appeared to promote HDM-induced allergic airway disease in male mice, its effects in female mice were not as clear-cut. Female LMAN1 KO mice showed significantly enhanced AHR compared with WT females, but did not exhibit concurrent increases in the HDM-induced inflammatory response, suggesting the contribution of other factors. It seems that the effects of LMAN1 in females may be targeted toward a specific cell type involved in AHR, such as airway epithelium or airway smooth muscle cells. Other studies support this conclusion by showing that, while inflammation often contributes to AHR, the 2 do not necessarily correlate. AHR is generally recognized as having two components: first, a fixed/chronic component, which reflects differences in airway structure due to remodeling or genetic predisposition, and secondly, a variable component, which fluctuates based on allergen exposure and inflammation.^34^ Intrinsic differences that comprise the chronic component have been identified in asthmatic ASM, such as enhanced activity of contractile proteins and increased shortening velocity.^34^^,^^35^ Thickening of the smooth muscle layer, the inner epithelial layer, and the outer wall of the airways is also strongly correlated with resistance.^36^ The uncoupling of inflammation and AHR is further demonstrated by *in vivo* research showing that strain-specific differences in mouse AHR are not linked with inflammation and instead correlate with other factors, such as ASM shortening velocity and expression of genes encoding G protein-coupled receptors, which regulate smooth muscle contraction.^37–39^ Thus, it is quite possible that LMAN1 acts through a specific mechanism in female mice to regulate ASM function without corresponding effects on inflammation. As previously mentioned, it is not likely that LMAN1 is simply affecting lung structure as no differences in lung function are observed in the absence of LMAN1 at baseline (Fig. S1).

Our research brings up the key question of what molecular mechanism LMAN1 might utilize to regulate responses to HDM in a sex-dependent manner. In the current work, we performed RNA sequencing in MTECs from WT and LMAN1 KO mice and used pathway analysis to identify areas that differ in expression between males and females of each genotype. Interestingly, we identified several sex-dependent trends found only in KO, but not WT, MTECs, demonstrating that loss of LMAN1 alters expression of these pathways. For example, the steroid hormone biosynthesis pathway was enhanced in KO males compared with KO females, an interesting area for future investigation given the key role of steroid hormones in regulating immune responses. Cortisol, a steroid hormone produced by the adrenal gland, is known for its ability to inhibit inflammation and is thus used as a therapy for allergic asthma, autoimmune diseases, and graft versus host disease.^40^ The sex steroid hormones could also potentially be implicated. Estrogen in particular has complex effects on AHR that do not parallel its enhancing effect on the immune response, suggesting that changes in estrogen signaling might explain the divergent trends in AHR in LMAN1 KO mice.^31^^,^^41^^,^^42^ In addition to affecting steroid hormone biosynthesis, loss of LMAN1 in MTECs sex-dependently influenced the pathway of adrenergic signaling. While adrenergic signaling is most recognized for contributing to AHR by regulating airway smooth muscle cell contraction, some studies have also shown that adrenergic signaling solely within the airway epithelial compartment is sufficient to influence AHR.^43^^,^^44^ Sex-dependent trends in adrenergic signaling have indeed been reported in the context of cardiovascular disease,^45^^,^^46^ and SNPs in the β2-adrenergic receptor have also been linked in a sex-dependent manner with persistence of asthma symptoms.^47^ Other pathways highlighted by our analysis include ABC transporters, Wnt signaling, and miRNAs in cancer, all of which warrant further investigation in relation to LMAN1.

In WT male and female MTECs, we conversely identified several pathways with sex-dependent trends in expression that differed from KO MTECs. WT females showed enhancement of relaxin signaling compared with WT males. Relaxin, a peptide hormone produced by the corpus luteum in females and the prostate in males, is known to exert antifibrotic effects and can partially reverse features of airway remodeling in mice with allergic asthma.^48^^,^^49^ Mice lacking relaxin show enhanced lung fibrosis and AHR both at baseline and in response to allergen, suggesting that sex-dependent modulation of this pathway could possibly explain the effects of LMAN1 on AHR.^50^ Another pathway found to be differentially regulated in WT males vs. females was PI3K-AKT signaling. In airway smooth muscle cells, PI3K promotes AHR by signaling through mTOR and AKT to promote ASM proliferation, and pharmacological inhibition of PI3K has been shown to prevent fibrosis and airway remodeling.^51^^,^^52^ Interestingly, the PI3K/PTEN axis has also been shown to be important within airway epithelial cells, with PI3K expression increasing and PTEN levels decreasing with exposure to allergen in this cell type, concurrent with the effects on AHR.^53^ PI3K signaling furthermore regulates activation of NF-κB and MAPKs, factors we know to be influenced by LMAN1, suggesting a potential area of crosstalk to explore.^52^^,^^54^ The AGE-RAGE pathway was also differentially altered in LMAN1 KO MTECs in a sex-dependent manner. Given that RAGE is currently being explored as a therapeutic target for asthma,^55^^,^^56^ further studies will be needed to elucidate how this pathway could be regulated sex-dependently in response to HDM, and how it could be influenced by loss of LMAN1.

One limitation of this work is that we did not control for effects of endogenous hormone levels, which vary throughout the estrous cycle in females. In subsequent studies, controlling for this factor may help to further define the role of LMAN1 in the allergic response in females. Experiments performed in WT versus LMAN1 KO mice with ovariectomy would also confirm the role of female sex hormones. Other future areas of research for our lab include studying the *in vivo* effects of LMAN1 KO in specific cell types through the use of conditional gene trap LMAN1 mice available at Jackson Laboratory/Mutant Mouse Resource & Research Centers (MMRRC). This would allow us to explore possible sex-dependent effects of LMAN1 in different compartments. These studies may help to clarify how LMAN1 acts as a negative regulator in DCs in vitro yet exerts sex-dependent effects in vivo.

In summary, our research has confirmed a critical role for LMAN1 in modulating HDM-induced inflammation. Our experiments demonstrate that LMAN1 could serve as a promising therapeutic target due to its ability to regulate the Th2 response as well as AHR. Our findings of sex-specific mechanisms suggest that LMAN1 should potentially be targeted through different methods in males and females. Uncovering the mechanisms of LMAN1 signaling and understanding its effects in different contexts may open possibilities for targeting this receptor with therapeutics that help address the unmet need in treatments for severe asthma.

## Supplementary Material

vkaf126_Supplementary_Data

## Data Availability

The RNA sequencing data that support the findings of this study are openly available at the Gene Expression Omnibus (GEO) Database under accession number GSE294581. All other data is described within the manuscript and Supplemental Information.
