# Supplementary material for: Sex-dependent influence of LMAN1 on allergen-induced airway hyperresponsiveness
Source: J Immunol. 2025 Jun 15;214(9):2397–407. doi: 10.1093/jimmun/vkaf126 (PMC12353830; doi:10.1093/jimmun/vkaf126)
Supplement: vkaf126_Supplementary_Data [file vkaf126_supplementary_data.zip › vkaf126_Supplementary_Data/JIMMUN-24-00497-s01.pdf]

UNIVERSITY OF CENTRAL FLORIDA

Justine Tigno-Aranjuez, Ph.D.
Associate Professor of Medicine
Immunity and Pathogenesis Division
Burnett School of Biomedical Sciences
UCF College of Medicine
BSBS Bldg. Rm 338 (office), Rm 370 (lab)
6900 Lake Nona Blvd,
Orlando, FL 32827
Office: (407) 266-7142
Lab: (407) 266-7143

April 8, 2025

To the Editors of the Journal of Immunology:

We would like to submit the revision for our manuscript entitled "Sex-dependent Influence of LMAN1 on Allergen-induced Airway Hyperresponsiveness" for consideration for publication in the Journal of Immunology as a full-length article.

Our previous work published in Cell Reports was the first description of LMAN1 (also known as ERGIC-53) as a *bona fide* cell surface receptor for the recognition of house dust mite. Prior to this, LMAN1 had only been considered as an internal cargo receptor for a select subset of glycoproteins. The current work submitted to JI is the follow-up to this initial work, investigating the role of LMAN1 in an *in vivo* model of house dust mite (HDM) - induced asthma. Not only do we confirm the involvement of LMAN1 in the sensing of HDM *in vivo*, but also discover that LMAN1 influences airway hyperresponsiveness in a sex-dependent manner. Such findings open the possibility for (a sex-dependent) therapeutic targeting of this receptor.

Main critiques of the initial submission included the lack of a clear mechanism for the observed sex-specific differences, a lack of connection between the presented changes in sex hormone receptor isoforms and disease, and unclear statistical analysis. In this revision, we have included additional data on early cytokines influenced by LMAN1 in a sex-dependent manner and RNA sequencing and pathway analysis data of primary airway epithelial cell cultures from WT and LMAN1 KO mice of both sexes. We have removed the data on sex-hormone receptors which was found to be premature. Lastly, we have simplified and clarified statistical analysis of the data. This new manuscript now includes 6 Main Figures and 8 Supplemental Figures and a revised discussion section.

This is the first such account of an *in vivo* role for LMAN1 in responding to allergens and the first to show a sex-dependent effect of LMAN1 on airway hyperresponsiveness. Given the scope and novelty of this work, we believe that JI is an appropriate journal for the manuscript. We hope that you agree and look forward to your response.

Sincerely,

JTaranjuez

Justine T. Tigno-Aranjuez
