# Supplementary material for: Sex-dependent influence of LMAN1 on allergen-induced airway hyperresponsiveness
Source: J Immunol. 2025 Jun 15;214(9):2397–407. doi: 10.1093/jimmun/vkaf126 (PMC12353830; doi:10.1093/jimmun/vkaf126)
Supplement: vkaf126_Supplementary_Data [file vkaf126_supplementary_data.zip › vkaf126_Supplementary_Data/JIMMUN-24-00497-s02.pdf]

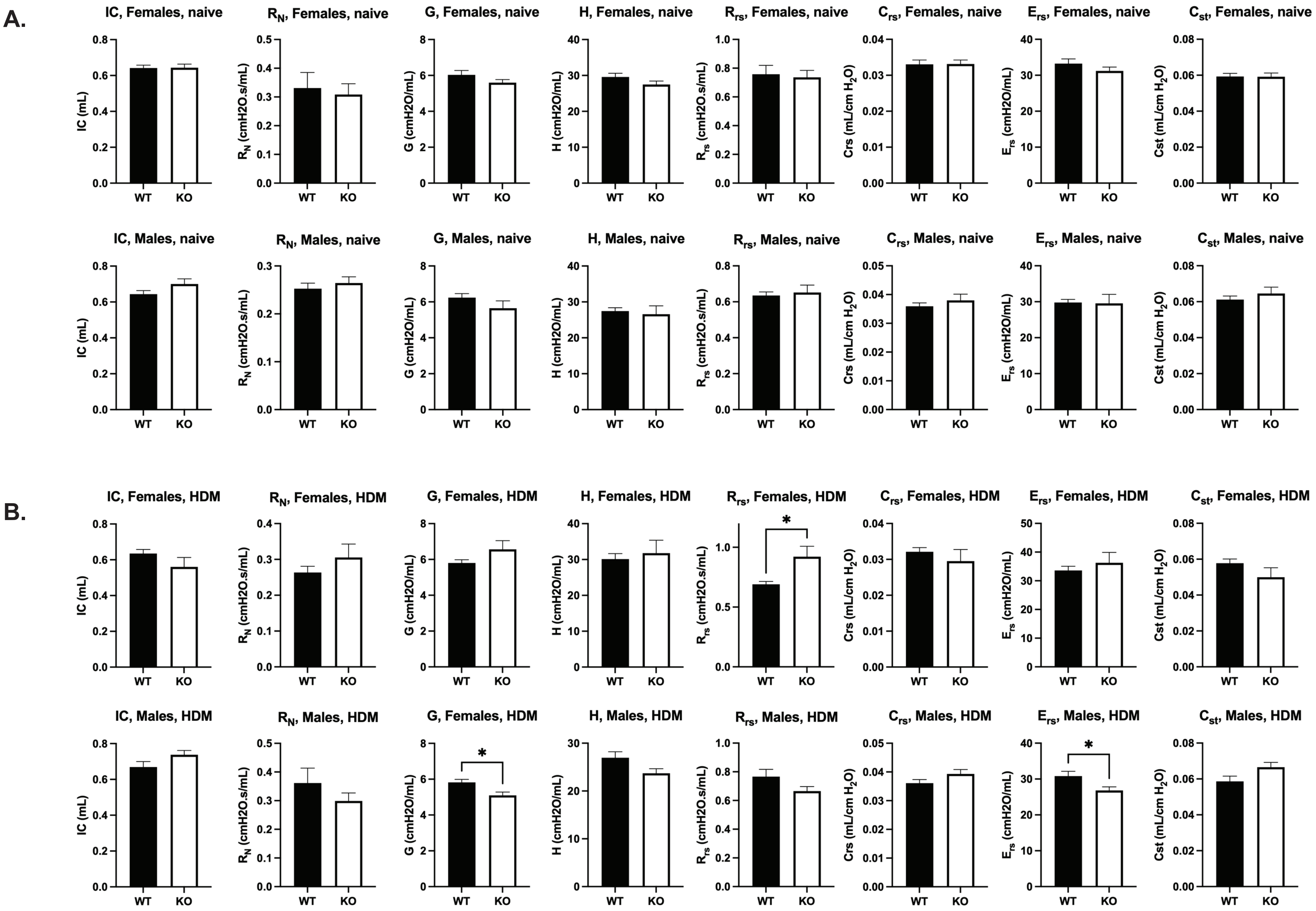

Supplemental Figure 1. Naive WT and LMAN1 KO mice of both sexes have similar baseline lung function which is altered when subjected to an HDM asthma model. Female and Male WT and LMAN1 KO mice were left untreated (**A**) or subjected to an HDM asthma model (**B**). After treatment, mice were anesthetized, cannulated and connected to a FlexiVent mechanical ventilator for analysis of baseline lung mechanics. The following parameters were analyzed: IC (inspiratory capacity), R_N (total resistance), G (tissue damping), H (tissue elastance), R_{rs} (total resistance), C_{rs}(system complance), E_{rs}(system elastance), C_{st} (static complance). An unpaired two-tailed Student's t-test was performed to assess significance between WT and KO groups. * = $p<0.05$ with n=6-13/group.

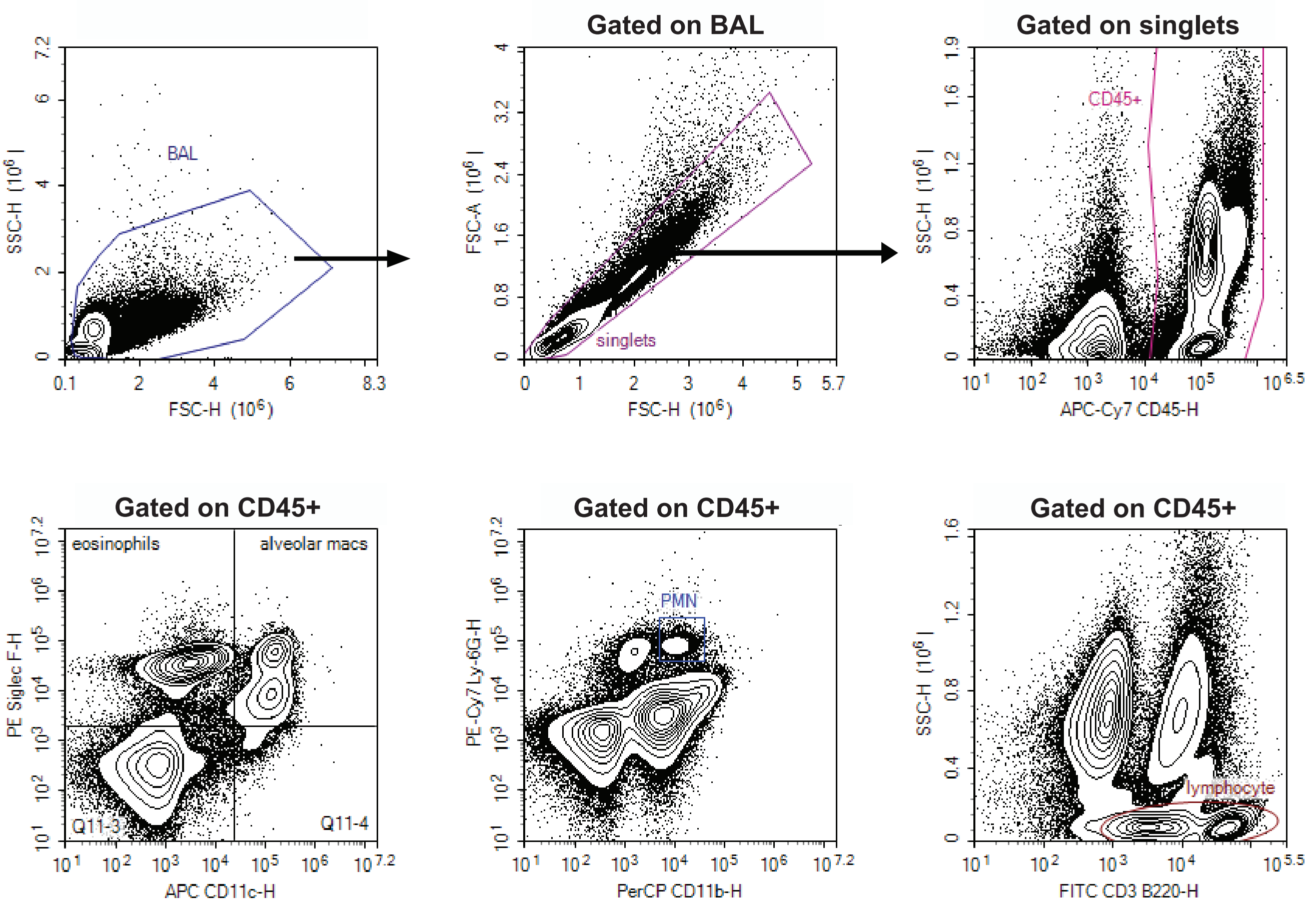

Supplemental Figure 2. Gating strategy for BAL staining. BAL samples were collected on day 14, and one million cells were stained with a panel of the following antibodies: FITC anti-mouse CD3, FITC anti-mouse B220, PE anti-mouse Siglec F, PerCP anti-mouse CD11b, PE-Cy7 anti-mouse Ly-6G, APC anti-mouse CD11c, and APC-Cy7 anti-mouse CD45. Eosinophils were classified as CD45+CD11c−Siglec-F+ cells, alveolar macrophages as CD45+CD11c+Siglec-F+ cells, neutrophils as CD45+CD11b+Ly-6G+ cells, and lymphocytes as CD45+ CD3/B220+ cells.

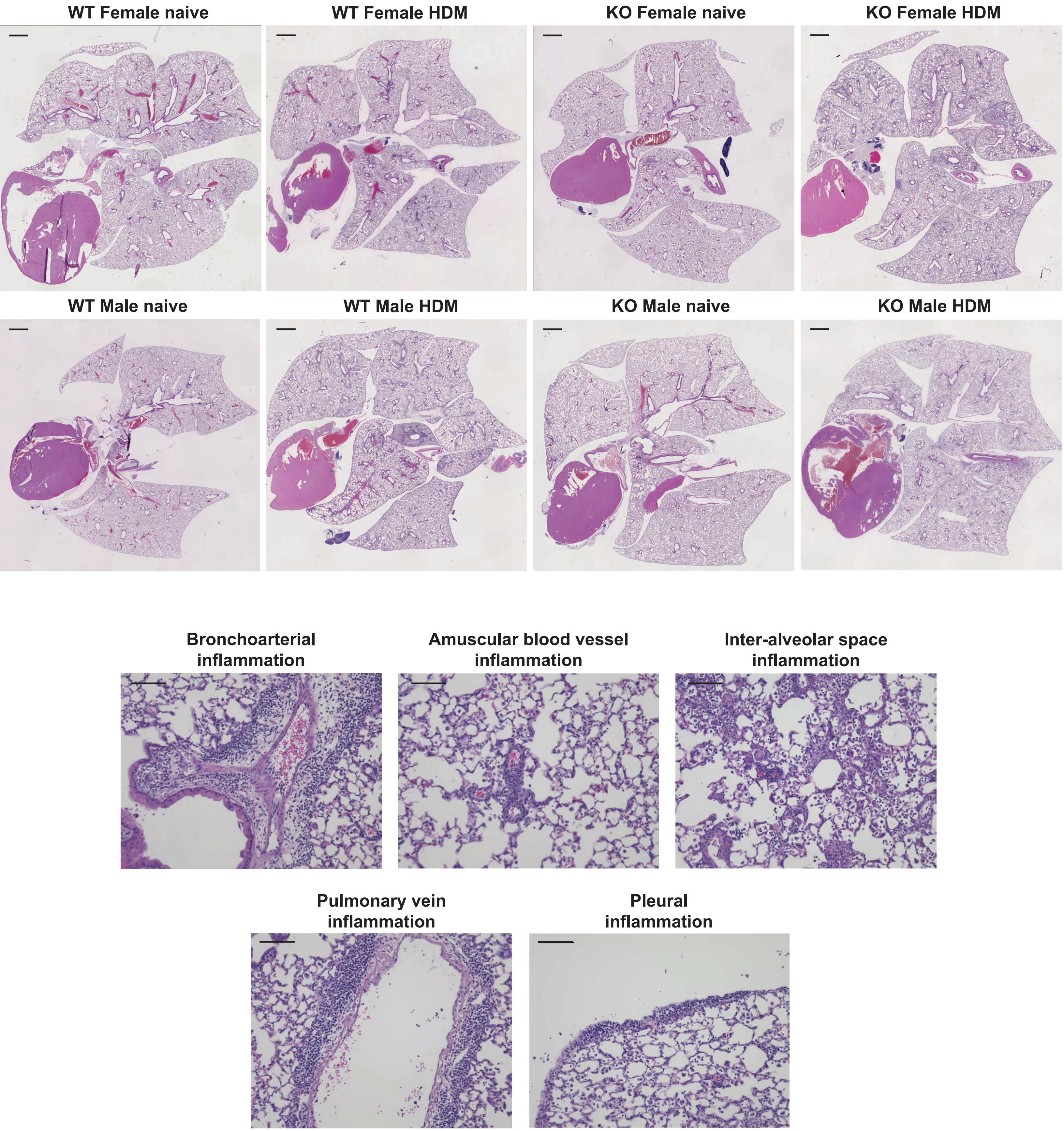

Supplemental Figure 3. Representative whole lung histology for naive and HDM-treated WT and LMAN1 KO mice. On day 14, mice were euthanized and lungs were fixed (inflated) with 10% buffered formalin. Fixed tissues were embedded in paraffin, sectioned, and stained with hematoxylin and eosin. An inflammatory index was calculated for each mouse and was based on the sum of individual scores for bronchoarterial inflammation, amuscular blood vessel inflammation, inter-alveolar space inflammation, pulmonary vein inflammation, and pleural inflammation. Representative examples for each of these types of inflammation are shown. (2x magnification, Scale bar = 1mm; 20x magnification, Scale bar = 100μm)

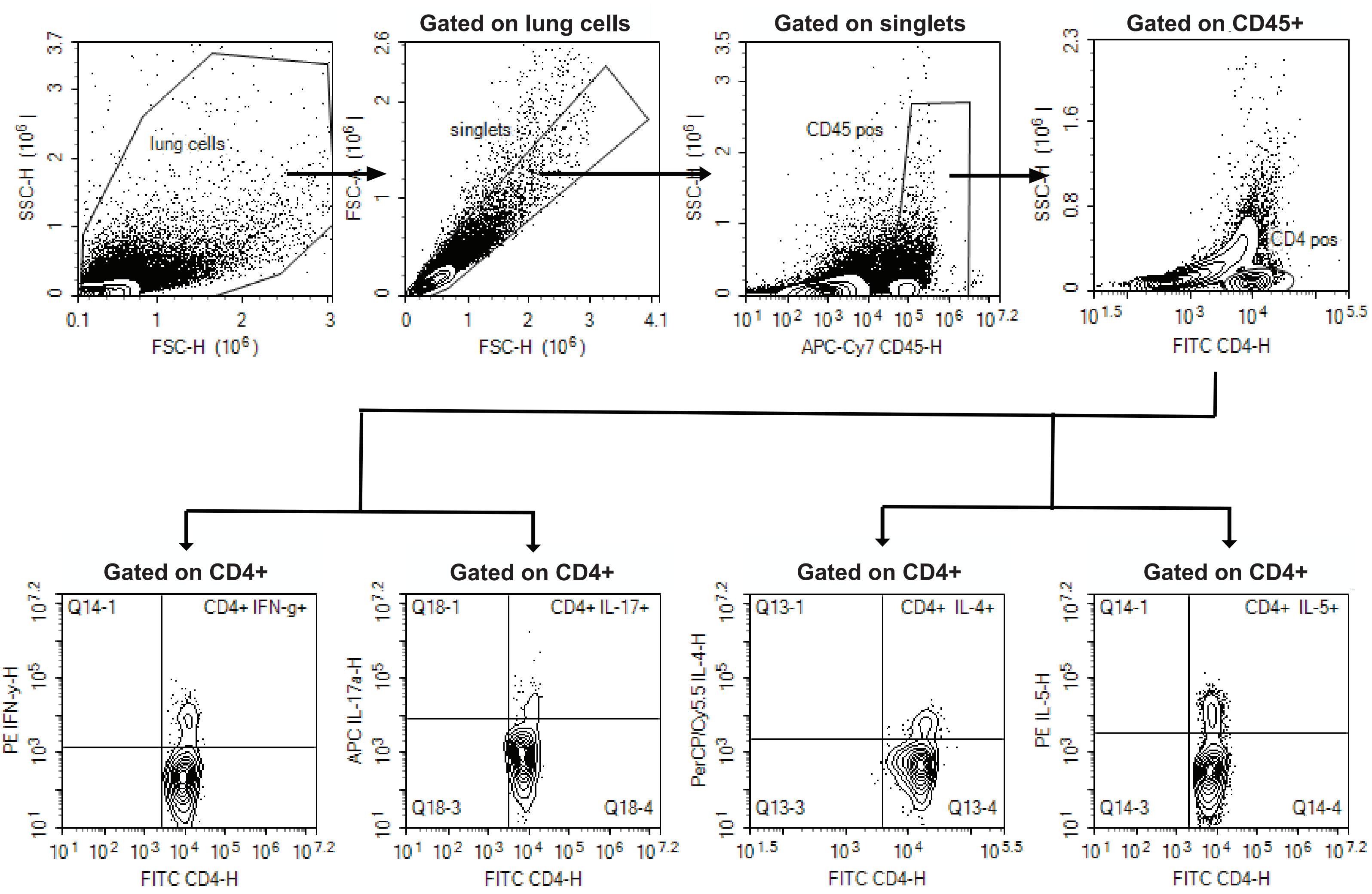

Supplemental Figure 4. Gating strategy for Intracellular Cytokine Staining (ICS). On day 14 of an HDM asthma model, mice were euthanized and the right middle lobe of the lung was dissociated by enzymatic digestion and mechanical dissociation and two million cells were used for ICS. Cells were stimulated with PMA + ionomycin in the presence of protein transport inhibitors and afterwards, cells were stained using the following antibodies. For cell surface staining, APC-Cy7 anti-mouse CD45 and FITC anti-mouse CD4 were used. Cells were then fixed and permeabilized and were stained intracellularly using PE anti-mouse IFN-γ and APC anti-mouse IL-17a (panel 1) or PerCP/Cy5.5 anti-mouse IL-4 and PE anti-mouse IL-5 (panel 2). Th1: CD45+ CD4+ IFN-γ+, Th17: CD45+ CD4+ IL-17+ and Th2: CD45+ CD4+ IL-4+ IL-5+

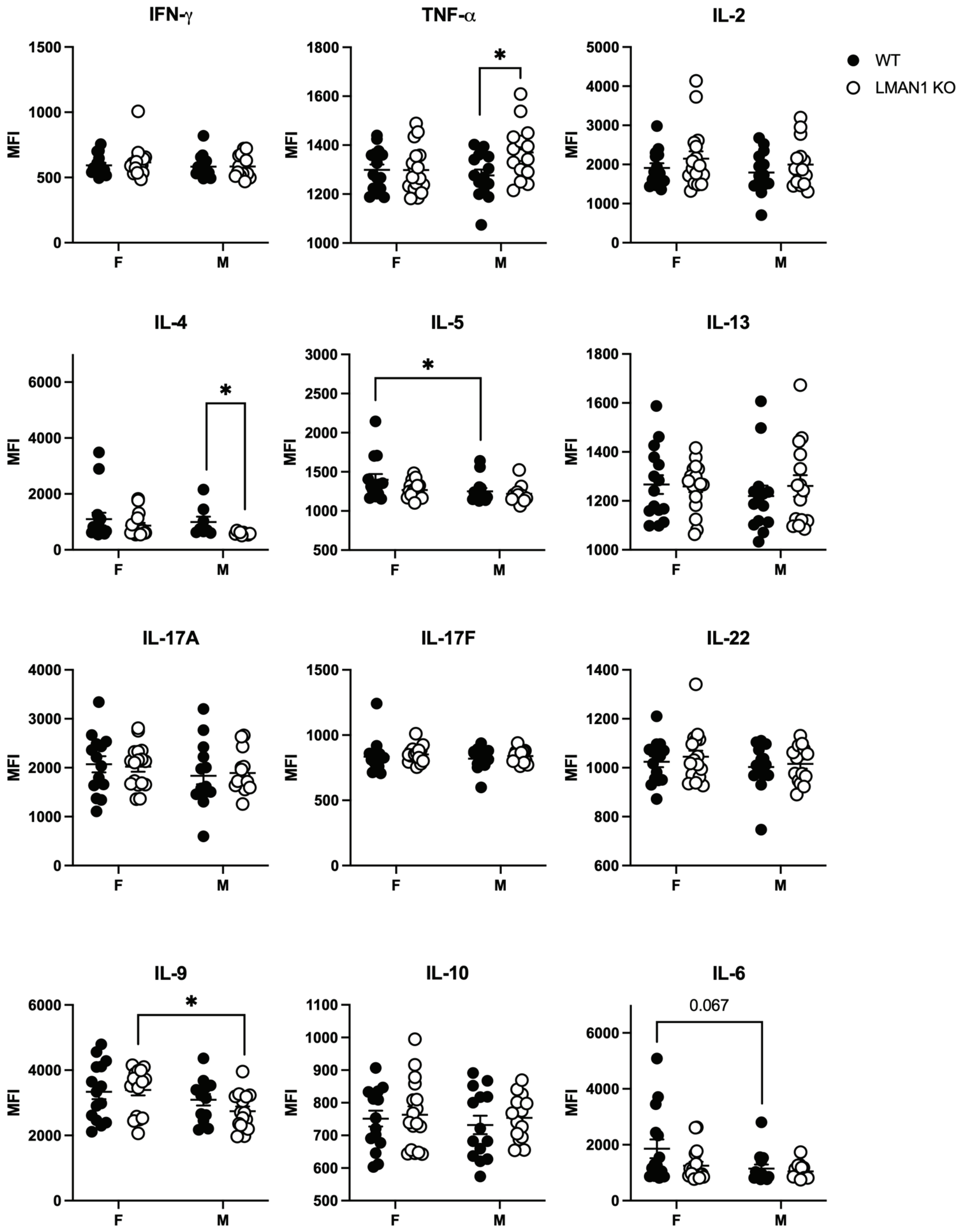

Supplemental Figure 5. Multiplex analysis of Th cytokines. On day 14 of a HDM asthma model, mice were euthanized and the lower right lung lobe was homogenized in tissue protein extraction reagent (TPER) supplemented with protease inhibitors. Lysates were collected, standardized and subjected to a bead-based multiplex assay (Legendplex) was used to assess production of various Th cytokines. Results were analyzed using a two-way ANOVA with multiple comparisons. * = $p<0.05$

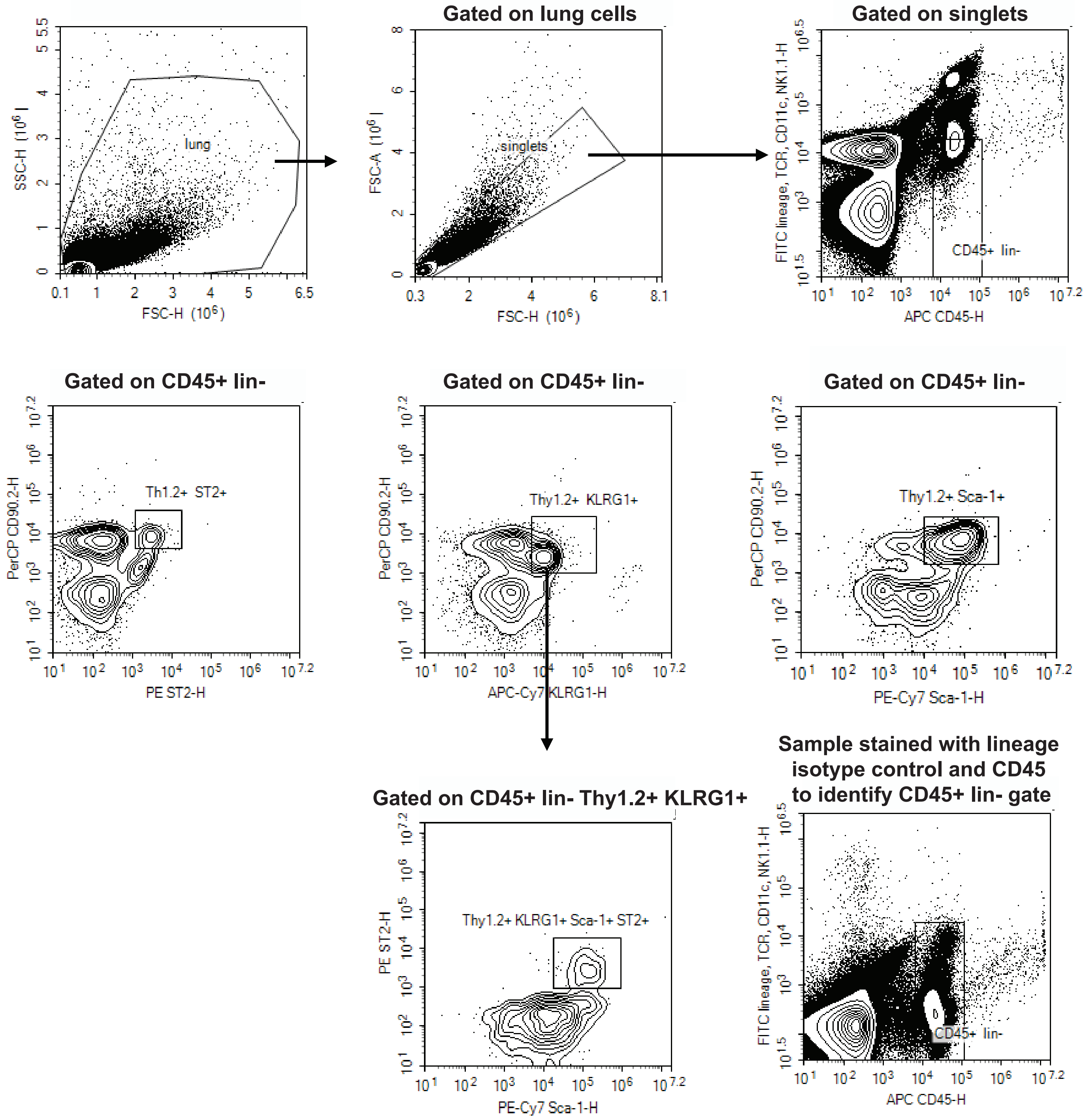

Supplemental Figure 6. Gating strategy for ILC2 staining. On day 14, the right middle lobe of the lung was dissociated by enzymatic digestion and mechanical dissociation and two million cells were stained with a panel of antibodies including FITC anti-mouse lineage (lineage cocktail of FITC labelled anti-mouse CD3ε, anti-mouse Ly-6G/Ly-6C, anti-mouse CD11b, anti-mouse CD45R/B220, anti-mouse TER-119 plus FITC anti-mouse TCRβ, FITC anti-mouse TCRδγ, FITC anti-mouse CD11c, FITC anti-mouse NK1.1), APC anti-mouse CD45, APC/Cy7 anti-mouse KLRG1, PerCP anti-mouse Thy1.2, PE anti-mouse ST2, and PE-Cy7 Sca-1. CD45+ Lin- cells were gated as shown above, using a sample stained with CD45 and a lineage isotype control antibody as a guide. ILC2s were delineated as the following four subsets: CD45+Lin-Thy1.2+ST2+ cells, CD45+ Lin-Thy1.2+KLRG1+ cells, CD45+Lin-Thy1.2+Sca-1+ cells, and CD45+ Lin-Thy1.2+KLRG1+Sca-1+ST2+ cells.

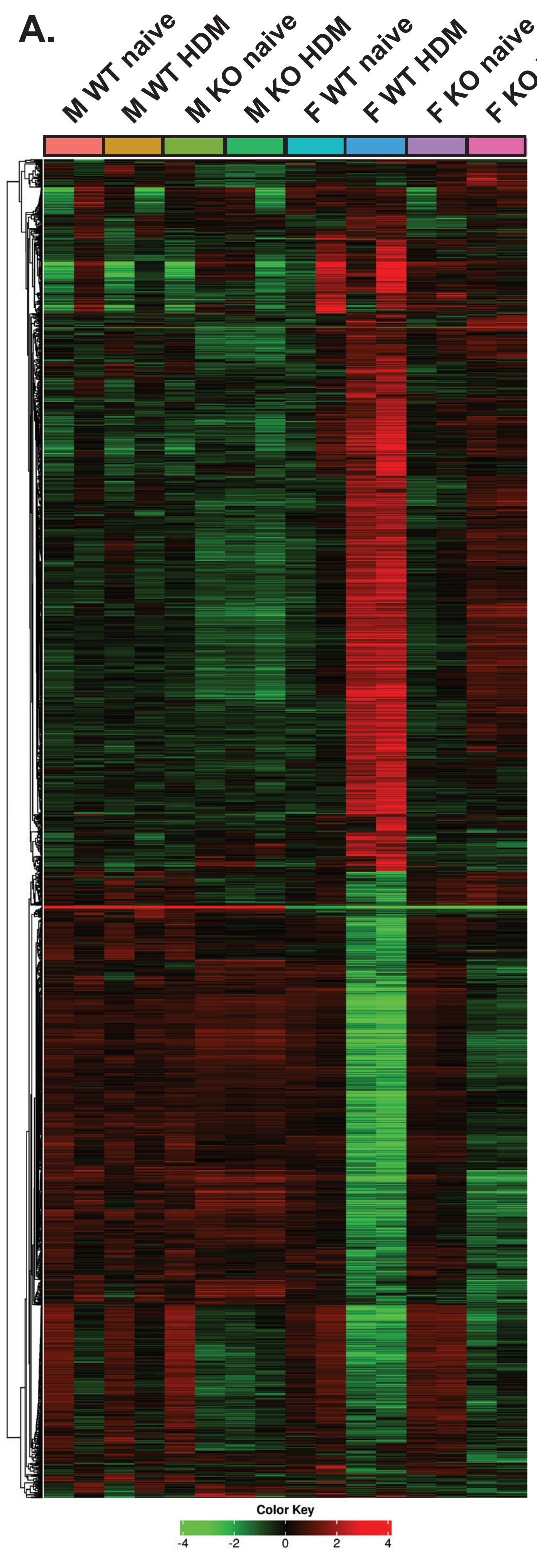

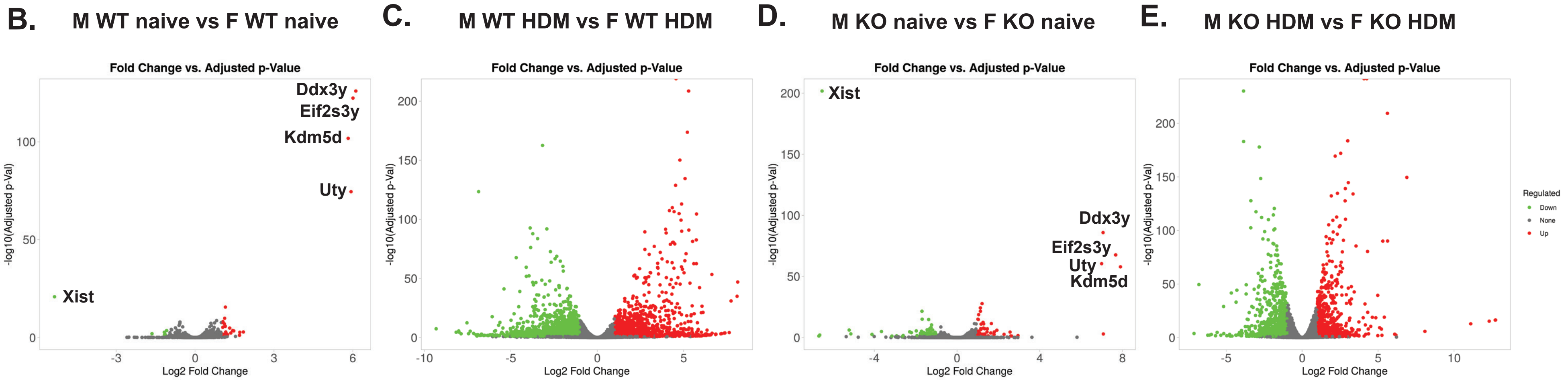

Top 5 differentially expressed genes

Gene Symbol	Log2FC	Adj. p-value
Ddx3y	6.11504637	1.17E-126
Eif2s3y	6.00739851	4.68E-123
Kdm5d	5.82427086	1.72E-102
Uty	5.93245707	3.04E-75
Xist	-5.3475638	1.42E-21

Top 5 differentially expressed genes

Gene Symbol	Log2FC	Adj. p-value
Xist	-6.5249755	1.87E-202
Ddx3y	7.06103294	1.18E-86
Eif2s3y	7.6700834	2.27E-68
Uty	6.99248399	3.30E-61
Kdm5d	7.90286886	1.37E-58

Supplemental Figure 7. RNA sequencing of murine tracheal epithelial cells (MTECs). MTECs were isolated from WT and LMAN1 KO mice (pooling 3 tracheas / 2 conditions). MTECs were plated on transwell inserts coated with 3T3 cell-derived basement membrane. These cells were allowed to expand submerged and fully differentiate under air liquid interface (ALI) conditions (around 12-14 days). Differentiated MTECs were left unstimulated or stimulated with 20µg/ml HDM extract overnight prior to harvesting RNA for RNA sequencing. **A**. Heirarchical clustering analysis of all groups. Volcano plots indicating differentially expressed genes for different comparisons: **B**. Male WT naive vs Female WT naive, **C**. Male WT HDM vs Female WT HDM, **D**. Male KO naive vs Female KO naive, **E**. Male KO HDM vs Female KO HDM. For comparisons without enough DEGs for pathway analysis, the top 5 DEGs are shown.

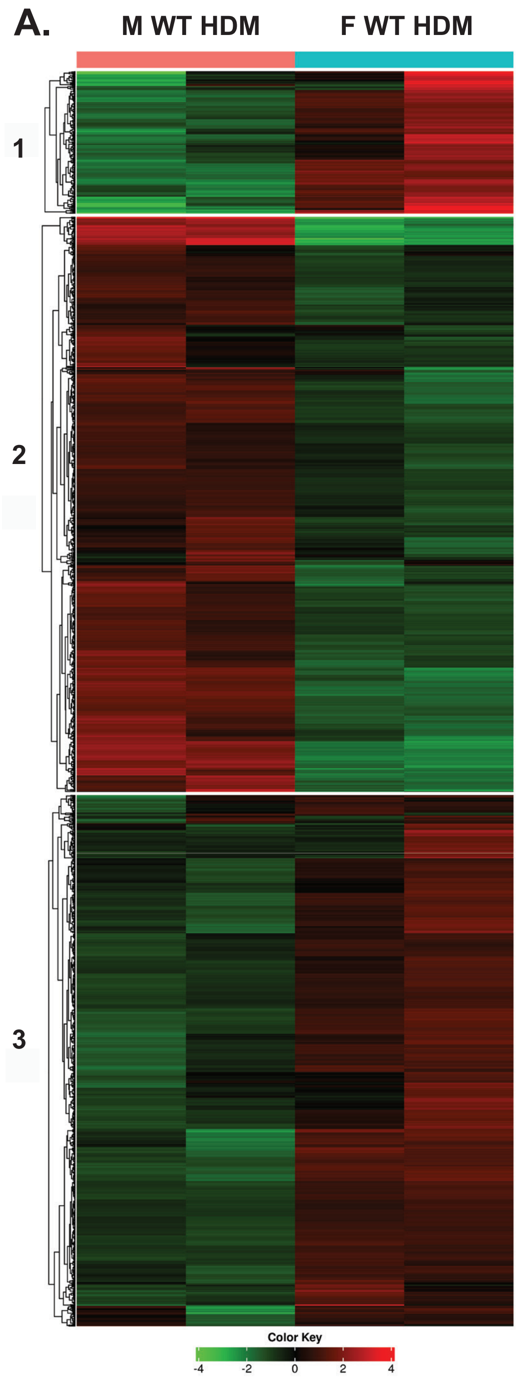

Group	Pathway	Fold Enrichment	FDR
1	Protein digestion and absorption	11.0484214	1.21E-08
1	ECM-receptor interaction	8.9104456	2.11E-06
1	Focal adhesion	4.49502248	3.48E-05
1	Relaxin signaling pathway	5.86897275	3.48E-05
1	AGE-RAGE signaling pathway in diabetic complications	6.26668702	3.48E-05
1	PI3K-Akt signaling pathway	3.39333333	1.09E-04
1	Proteoglycans in cancer	3.49500624	3.31E-03
1	Cytokine-cytokine receptor interaction	3.65556743	3.75E-03
1	TGF-beta signaling pathway	4.71296296	1.17E-02
1	Melanoma	5.27212806	1.43E-02
2	Drug metabolism-cytochrome P450	10.634378	2.61E-18
2	Metabolism of xenobiotics by cytochrome P450	9.50447531	1.71E-15
2	Retinol metabolism	8.36170848	2.26E-09
2	Drug metabolism-other enzymes	5.57447232	6.52E-09
2	Chemical carcinogenesis-DNA adducts	7.62391068	7.14E-09
2	Glutathione metabolism	5.65555556	2.96E-08
2	Taurine and hypotaurine metabolism	11.5205761	1.76E-06
2	Metabolic pathways	1.5429343	7.77E-06
2	Bile secretion	4.32021605	8.25E-04
2	Pentose and glucuronate interconversions	6.09912854	6.18E-03
3	Cytokine-cytokine receptor interaction	3.33120298	3.90E-07
3	Calcium signaling pathway	2.86743618	9.95E-05
3	Cell adhesion molecules	3.35269461	1.96E-04
3	PI3K-Akt signaling pathway	2.19449102	2.20E-04
3	ECM-receptor interaction	3.928939	2.20E-04
3	Malaria	5.58782435	2.20E-04
3	Focal adhesion	2.51936589	3.45E-04
3	Viral protein interaction with cytokine and cytokine receptor	3.9443466	1.03E-03
3	Neuroactive ligand-receptor interaction	2.82332178	3.76E-03
3	Amoebiasis	2.86205638	7.66E-03

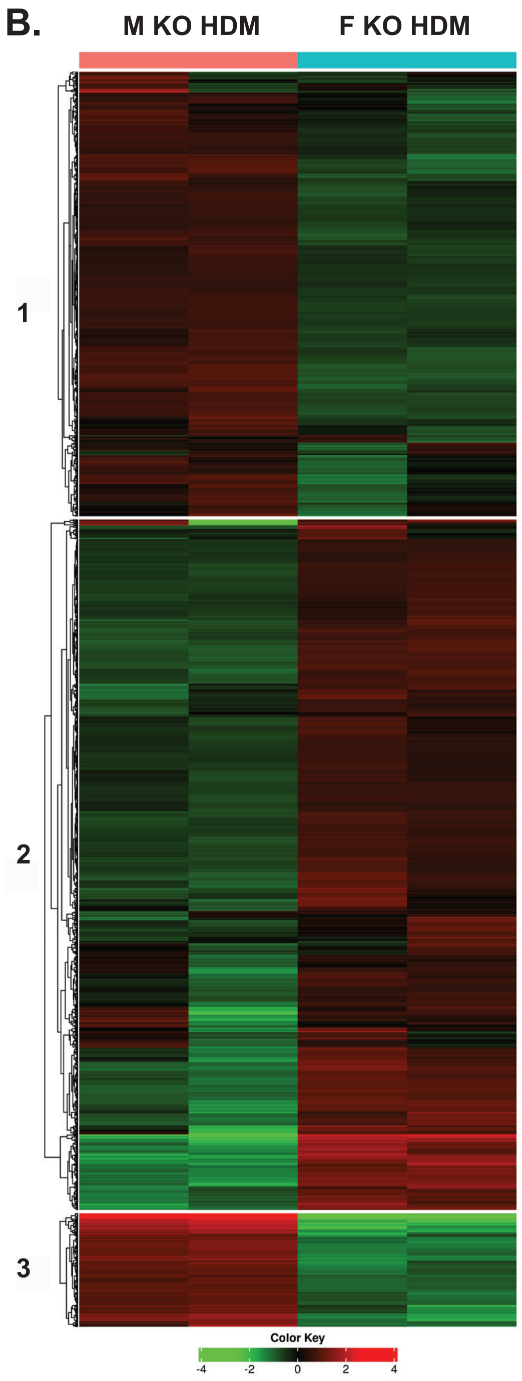

Group	Pathway	Fold Enrichment	FDR
1	Drug metabolism-cytochrome P450	7.65546219	1.19E-06
1	Metabolism of xenobiotics by cytochrome P450	6.16171347	1.16E-04
1	Retinol metabolism	6.45929622	5.17E-04
1	Taurine and hypotaurine metabolism	10.5998707	5.84E-04
1	Metabolic pathways	1.49574983	2.38E-03
1	Chemical carcinogenesis-DNA adducts	5.40385566	3.57E-03
1	Glutathione metabolism	3.69102641	2.40E-02
1	Cytokine-cytokine receptor interaction	2.44323261	3.82E-02
1	Drug metabolism-other enzymes	3.33383031	3.97E-02
1	Steroid hormone biosynthesis	4.78466387	8.88E-02
2	ECM-receptor interaction	3.96086957	1.45E-04
2	Calcium signaling pathway	2.54173448	9.69E-04
2	Neuroactive ligand-receptor interaction	2.93397746	3.94E-03
2	Cell adhesion molecules	2.73512732	4.73E-03
2	Adrenergic signaling in cardiomyocytes	2.60403189	4.81E-03
2	ABC transporters	3.60762313	3.99E-02
2	P53 signaling pathway	2.63382631	3.99E-02
2	Protein digestion and absorption	2.90141389	3.99E-02
2	Wnt signaling pathway	2.05581465	4.09E-02
2	MicroRNAs in cancer	2.0107557	4.09E-02

Supplemental Figure 8. Pathway analysis of RNA sequencing data derived from WT and LMAN1 KO MTECs. **A.**, **B.** K-means clustering was performed followed by enrichment analysis using KEGG pathways. The top10 pathways for every cluster including the fold enrichment and FDR are listed in the tables to the right of each cluster analysis. In red are pathways which are present in one comparison but absent from the other (present in **A** but absent in **B** and vice versa).
